# Effect of Immersive Virtual Reality on Post-Baccalaureate Nursing Students’ In-Dwelling Urinary Catheter Skill and Learning Satisfaction

**DOI:** 10.3390/healthcare10081473

**Published:** 2022-08-05

**Authors:** Chu-Ling Chang

**Affiliations:** Nursing Department, HungKuang University, No. 1018, Sec. 6, Taiwan Boulevard, Shalu District, Taichung City 43304, Taiwan; sxc46851@sunrise.hk.edu.tw; Tel.: +886-4-26318652 (ext. 3151)

**Keywords:** immersive virtual reality, post-baccalaureate nursing students, skill for managing female in-dwelling urinary catheters

## Abstract

A fundamental skill required from nursing students is how to manage the insertion of in-dwelling urinary catheters, and this skill is a core competency for nurses. However, practice with conventional test models is insufficient for learning this skill and leads to inadequate proficiency among students. To address this problem, this study created an immersive virtual reality (IVR) scheme, based on the theory of situated learning, to simulate clinical situations. Innovative approaches were adopted to design clinical cases, construct three-dimensional environments, design character dialogs, and integrate artificial intelligence voice recognition. The effect of these design elements on students’ in-dwelling urinary catheter skills and learning satisfaction was explored. First, nursing experts assessed the quality of the IVR scheme. Over a 4-week period, 43 students in a post-baccalaureate nursing program used conventional test models to practice the management of in-dwelling urinary catheters in female patients, and their learning was supplemented by at least two practice sessions with IVR. Data were collected from in-class observation records, a questionnaire survey on student satisfaction, and focused group interviews. The results showed that the participating students were highly satisfied with the IVR scheme and stated that it provided a pleasurable learning experience and exerted a positive impact on them. The IVR scheme provided situations closely resembling real clinical environments, helping the students to memorize the steps for catheter management. The students also noted that the IVR scheme should incorporate other nursing skills, such as empathetical and solicitous care and patient companionship. This enables nursing students to fulfill their role and care for patients in clinical settings.

## 1. Introduction

When using immersive virtual reality (IVR), users can see, hear, and interact with virtual environments, enabling them to completely immerse themselves in simulated scenarios [1]. New IVR technologies offer unprecedentedly vivid environments and realistic and immersive experiences [2]. The phrase “immersive experience” refers to an experience in which users fully immerse themselves in a simulated situation. That is, digital technology or an environment that employs augmented reality (AR), virtual reality (VR), mixed reality (MR), or projection technology is employed to create a near-realistic situation and the users become fully immersed in the situation, connecting and resonating with it. Immersive learning involves guiding learners into a fully immersive environment, reducing external interferences. In this immersive experience, learners’ brains actively grasp and memorize information. Gamifying neonatal resuscitation training using immersive VR has effectively enhanced nursing students’ neonatal resuscitation knowledge, problem-solving skills, self-confidence, and learning motivations [3]. Interactive media have been widely studied and applied abroad. For example, one study investigated the use of interactive media for entertainment, specifically for providing theater audiences with a sense of realism [4]. Several studies evaluating the applications of IVR in medical care have yielded favorable results. For example, in one pediatric care study, researchers compared the use of IVR and kaleidoscopes in helping child patients to understand the treatment process before undergoing venipuncture, thus minimizing the patients’ pain and anxiety. The results indicated that compared with kaleidoscopes, IVR could more effectively minimize the patients’ pain and anxiety [5]. In another study, scholars used IVR to prepare child patients with cancer and their families for regular pediatric radiation therapy. The results of the study provided preliminary support for the use of IVR to achieve favorable clinical and surgical outcomes in pediatric populations [6]. Another study revealed that interactive media–based interventions are effective in treating depression [7]. Regarding medical or nurse education, IVR improved skills focusing on cooperative learning and reinforcement [8]. In one study, game-based VR was applied in tracheostomy care education for nursing students, to help the students hone their psychomotor skills [9]. Another study applied IVR in an interprofessional round training program for healthcare students. By participating in an IVR simulation program, the students gained a profound understanding of the distinct roles of the different members of interprofessional teams and how to interact with their team members. The IVR simulation program thus reduced the gap between nursing school and clinical settings [10]. IVR simulations designed to simulate teamwork enabled users to cooperate and interact with one another within the system [1]. According to one study, VR is an effective assistive tool that students can use to learn specific clinical skills, such as inserting urinary catheters [11]. In another study, IVR was used to teach undergraduate nursing students how to care for a patient with a foreign body in their right lung. The results revealed that the students proactively participated in the IVR simulation and expressed the opinion that the IVR-based teaching method was beneficial to their learning [12]. Another study used IVR to educate nursing students on simple triage in a simulated mass casualty incident, and the results revealed that the IVR training program was as effective as a clinical simulation [13]. In another study in which IVR was used to simulate a mass casualty incident, the sense of presence in an immersive scenario was determined to be correlated with immersion propensity [14]. Another study on health education focused on participants’ feelings when they used gamified two-dimensional, three-dimensional, and IVA-based educational tools to study clinical cases. The participants perceived the IVR-based tool to be the most conducive to improving their learning performance [15]. In a recent study evaluating VR simulation training for adult students, the students had high expectations regarding the VR simulation training and offered suggestions for improvement after completing the VR simulation training; these suggestions may serve as a reference for nursing educators in developing more effective VR-based training programs [16].

In Taiwan, the applications of interactive media have proliferated in recent years. One such application is in entertainment devices at theme parks, which enable visitors to immerse themselves in simulated environments [17]. One Taiwanese pilot study evaluated the use of interactive media in ecotourism for older adults, which yielded favorable results [18]. However, most applications of IVR in Taiwan have been limited to the fields of art, culture, and recreation. To create new experiences for users, IVR simulations should emphasize pleasure, inspiration, and creativity [19]. According to a survey of university-education personnel, antidrug teaching materials integrated with IVR technology exerted substantial effects on the behavior of high-school students and were more effective than conventional teaching methods [20]. In medical education in Taiwan, VR has been applied in pre-internship pharmaceutical education at teaching hospitals, specifically for the purposes of training new pharmacists in dispensing medicine. Participating in a VR-simulation training program enhanced participants’ motivation and learning performance and helped them to accumulate hands-on experience [21]. Clinical nursing supervisors in Taiwan have suggested that because VR is interactive and provides a sense of touch and movement when simulating the human body or sensory organs, it effectively piques the learners’ interest, thereby improving their learning performance and comprehension. IVR is suitable for helping nurse practitioners to hone their critical thinking skills, including those related to speculation, reasoning, and differentiation [22]. One medical center in Taiwan offered a flipped training course in which a VR-based lesson plan was used to help nurses practice placing peripherally inserted central catheters. The nurses were interested in the course and, using VR, were able to effectively learn and retain knowledge regarding the correct way to perform the procedure [23]. Nevertheless, there have been no previous studies conducted in Taiwan that explored the applications of IVR in university nursing programs. However, most university nursing students are not in-service students, and universities often experience difficulties in providing opportunities for nursing students to practice clinical skills in actual clinical settings. Researchers and practitioners must consider how to overcome this problem to help students hone their hands-on abilities.

The technique regarding how to place Foley catheters ins female patients is one of the most common and basic clinical nursing skills that every nursing student must have mastered before commencing clinical work. Moreover, according to the worksheet used in the present study, we discovered that the post-baccalaureate nursing students who participated in this study faced difficulties learning such skills, including “having to memorize the operational steps without a clinical scenario,” being “unable to memorize the steps,” and “having difficulty memorizing all the items involved in the technical operation.” One approach to helping students gain clinical experience, memorize operational steps, and overcome other difficulties in learning these skills involves the use of IVR. The research participants were students in a post-baccalaureate nursing program. Their difficulties lay in the students’ inability to install an in-dwelling urinary catheter completely and correctly, which necessitates numerous complicated steps. The research question of this study was whether employing IVR teaching could resolve their learning issues. The research objective was to explore the effect (i.e., solving the students’ problem and offering learning satisfaction) of IVR teaching on learning performance regarding in-dwelling urinary catheter skills.

## 2. Materials and Methods

### 2.1. Materials

The development of IVR teaching materials consists of writing simulation scenarios and dialogs, constructing three-dimensional scenes, and designing characters, as well as integrating artificial intelligence voice recognition capabilities and coordinating with media engineers to convert the developed content into engineering documents. Certain pieces of hardware, such as headgear and computers, were required to use the IVR platform.

### 2.2. Research Design

**Figure 1:**
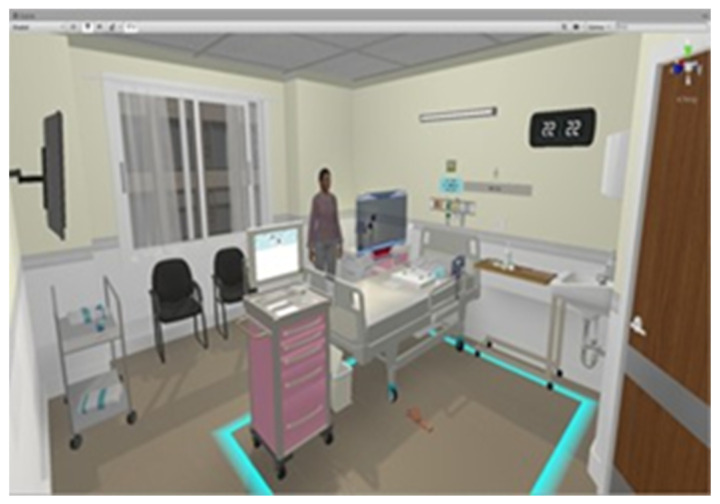
IVR simulation regarding how to place Foley catheters in female patients.

**Figure 2:**
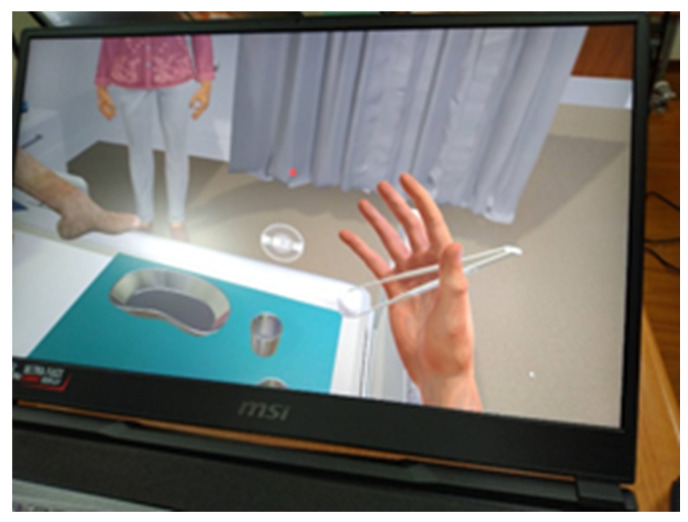
The participant’s viewpoint.

This study adopted a quasi-experimental design. The independent variable of this study was IVR teaching, while the dependent variable was learning performance. Learning performance consisted of solving students’ learning problems, providing learning satisfaction, and discerning their learning experiences and feelings toward their in-dwelling urinary catheter skills. We designed a clinical scenario and collaborated with an information technology engineering team to produce an IVR-based lesson plan on how to place Foley catheters in female patients (Figure 1 and Figure 2). We used a model to demonstrate how to place Foley catheters in female patients and used the IVR platform for additional demonstrations and explanations. The participants of this study were 43 freshman students of a postbaccalaureate nursing department, all of whom had earned bachelor’s degrees in non-nursing majors and were aged 23–38 years. All the participants were first-time learners of the technique for inserting in-dwelling urinary catheters. This study was approved by the Institutional Review Board, and all the participants were full-time students. The students were instructed on the goals of the VR teaching and research procedure and had signed informed consents before this study commenced. Students who were unable or unwilling to use the VR technology were excluded from this study, without repercussions on their grades. Over a 4-week period, each student used the model to study how to place Foley catheters in female patients and used the IVR platform to practice performing the procedure at least twice. We collected and statistically analyzed class observation records, satisfaction questionnaires, and focus group interviews. The measurement tool of this study was a learning satisfaction and learning impact questionnaire that was produced for this study. The questionnaire employed a 5-point Likert scale design and the questionnaire comprised 10 items. The questionnaire was administered after its reliability and validity were tested. The items were as follows:

1. Using VR can partially resolve the difficulties in learning in-dwelling urinary catheter skills.
2. Using VR helps me memorize the steps.
3. Using VR makes me feel as if I were in a clinical setting.
4. Using VR solves my learning issues.
5. Using VR improves my learning performance.
6. After using VR, I feel the VR is of good quality.
7. Using VR to practice is convenient.
8. I think that VR is a suitable assistive learning device for learning nursing skills.
9. Using VR to practice skills makes me uncomfortable.
10. In the future, I would like to use VR again to help my learning.

This study held focus-group interviews to understand students’ learning experiences and feelings. The interview was conducted 3 days after the students engaged in VR training. The interview outline consisted of the following examples: “Have you ever used VR?”, “How do you feel about using VR this time?”, “How does the VR application help or influence your skills in handling in-dwelling urinary catheters in female patients?” and “What are some suggestions you have for using VR?” The interview guidelines were inspected by teacher experts in the same department as the author. The focus group interview was hosted by the author. At the beginning of the interview, the interview rules were explained to the interviewees: “Each participant is allowed to express their opinions and must raise their hand to speak. Do not interrupt others when they are speaking and do not speak in an interrogating tone.”

### 2.3. Procedure

**Figure 3:**
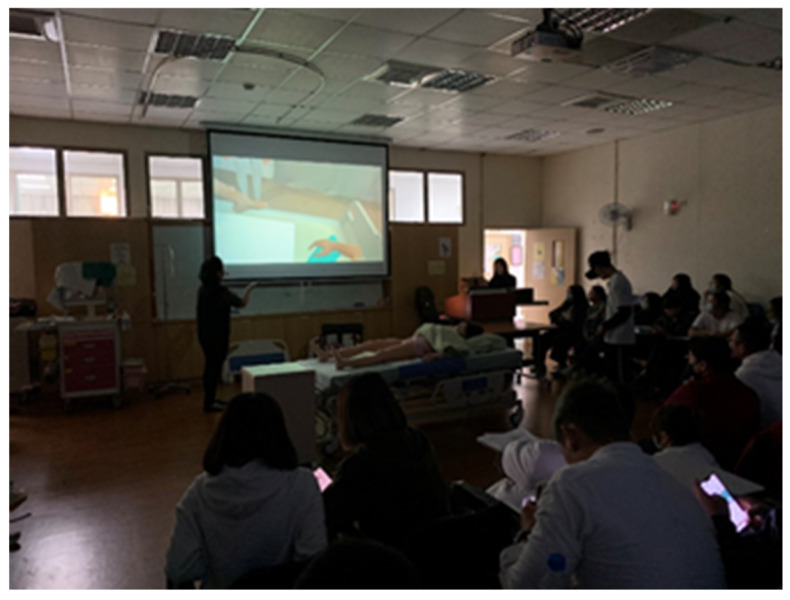
The researcher explaining IVR to the students.

**Figure 4:**
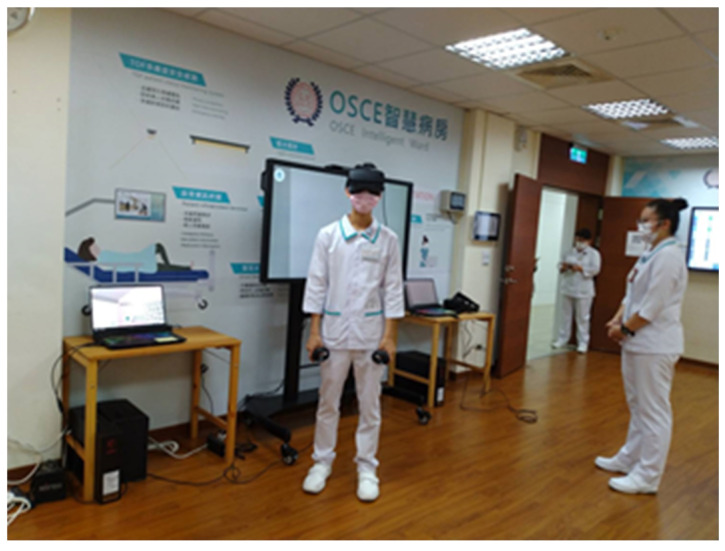
A student practicing using the IVR system.

The study process comprised three stages, namely, the pre-teaching, teaching, and post-teaching stages (Table 1). In the teaching stage, we demonstrated how to place Foley catheters in female patients in real life and by using the IVR platform (Figure 3). During the VR demonstration process, the view from the operator was projected onto a screen to enable the whole class to observe the VR operation process. Each student practiced the technique, using the VR system, at least twice (Figure 4).

### 2.4. Data Analysis

The analytical approach of this study involved a combination of qualitative and quantitative methods. We analyzed the qualitative and quantitative data collected via a learning satisfaction questionnaire survey and a focus group interview. Each item on the learning satisfaction questionnaire was scored on a 5-point Likert scale. We used Microsoft Excel to calculate the frequency of each score (as a percentage) and the mean score for each item. We recruited 11 of the students as voluntary participants for the focus-group interview. The interview outline includes the questions “Have you ever heard of or used VR?” and “How did you feel when you first used VR?” The interviewees were also asked about the effects of using the IVR system to practice how to place Foley catheters in female patients and their suggestions on how to improve the process. All the interviewees agreed to be voice recorded. The recording of each interview was transcribed for inductive content analysis. After the focus group interview, the recordings of the interview were transcribed. The author compiled and coded the transcripts and the transcripts were inspected by a teacher from the same department as the author. The students’ experiences and feelings about practicing using VR were discussed in terms of three topics and five categories.

## 3. Results

A total of 43 first-year students in a post-baccalaureate nursing program participated in the study. All the participants had a bachelor’s degree (100%), and their ages ranged from 23 to 40 years old (mean: 25 years). Nine (21%) of the participants were men, and 34 (79%) were women.

### 3.1. Learning Satisfaction

We evaluated the students’ satisfaction and experiences with using VR to learn how to place Foley catheters in female patients. The participants’ mean score on the learning satisfaction questionnaire was 4.37. Their mean scores on the VR scenario and content, the innovative teaching method, and the VR operation procedure were components of the satisfaction questionnaire and were 4.42, 4.40, and 4.42, respectively, indicating that most of the students were highly satisfied with the IVR-based lesson on how to position Foley catheters in female patients.

### 3.2. Effects of Using VR to Learn and Practice How to Insert Foley Catheters in Female Patients

Regarding the effects of using VR to learn how to position Foley catheters inside female patients, 25 (58.6%) of the students reported that the IVR platform helped them overcome the difficulties that they had experienced in learning the technique; the remaining 18 (41.4%) students’ responses were neutral. Regarding the statement, “Using VR can help me memorize the operation procedure,” six (13.8%) of the students strongly agreed, 21 (48.3%) agreed, 15 (34.5%) were neutral, and one disagreed. Regarding the statement “Using VR to practice makes me feel as though I were in a clinical setting,” two (4.6%) of the students strongly agreed, 20 (44.8%) agreed, 18 (41.4%) were neutral, 3 (6.9%) disagreed, and 3 (6.9%) strongly disagreed. A total of 39 (90.7%) of the students reported that they thought using VR to practice the technique was convenient; the remaining 4 (9.3%) reported that it was inconvenient. Regarding the suitability of using VR to learn how to position Foley catheters inside female patients, 30 (69.8%) of the students reported that it was suitable or extremely suitable, one (2.3%) reported that it was unsuitable, and 12 (27.9%) were neutral. Regarding whether the students would like to continue using VR to learn, 36 (82.8%) of the students said they would, and the remaining 7 (17.2%) said they would not. Overall, most of the students expressed a positive attitude toward using VR to learn.

### 3.3. Learning Experience and Feelings about the IVR Training

We divided the students’ experiences with and feelings toward using VR to learn how to place Foley catheters in female patients, as described in the focus group interview, into three themes and five categories (Table 2).

### 3.4. Pleasurable Experience

Most of the participants had not previously used VR. When they first used it, they felt a sense of novelty. They stated that during the IVR practice, they could see different scenarios; for example, one student said, “I was shocked because there were such scenes. I thought, wow, this world turned out to be fun” (Student 8). The students’ interest in using VR to practice was stimulated during the training, and some of the students even directly expressed the opinion that using VR to learn was fun (“It was kind of fun” (Student 5); “It was super fun” (Student 9)). While observing the students who were practicing using the IVR platform, we noted that most of the students were interested in VR and actively engaged in the IVR training.

### 3.5. Effective Learning

Some of the students reported that using VR to practice effectively helped them to learn how to place Foley catheters in female patients. Exposure to a simulated clinical scenario helped them memorize the steps involved in the procedure regarding the placement of Foley catheters in female patients. They were given a sense of the clinical and practical aspects of the procedure before starting a hospital internship, and they acquired a new understanding of the applications of technology in medicine.

#### 3.5.1. Being in a Clinical Scenario

Some of the students stated that using VR to practice was like being in an actual clinical setting. For example, students made the following statements: “I could feel a sense of presence, and the visual effect” (Student 1); “Back then, it was a more fragmented concept. Now, it’s real. It has the actual sense of clinic-ness” (Student 4); “It has a sense of body” (Student 9); “It has a sense of touch” (Student 2); and “It provides a sense of interaction” (Student 7).

#### 3.5.2. Memorizing the Steps of the Procedure Regarding the Placement of Foley Catheters in Female Patients

Many of the students maintained that using VR to practice how to place Foley catheters in female patients helped them to memorize the steps involved in the procedure. For example, students made the following statements: “It has the advantage of enabling me to memorize all the steps” (Student 7); “It involves a step to memorize the procedure” (Student 12); “It enables a student to become familiar with a technique. I feel it consists of steps to help students familiarize themselves with the technique” (Student 5); “So far, it is for practice” (Student 10). “I use it to memorize the steps” (Student 11); “Using VR in our study prevents us from making numerous mistakes when we enter a clinical setting. I’m grateful for this learning opportunity” (Student 6); “The greatest help VR provides is the procedure” (Student 2); and “It enabled me to become familiar with the procedure. By continually operating the system, I came to see what I was doing” (Student 3). The technique of placing Foley catheters in female patients involves many operational steps, and the entire procedure must be performed in a sterile environment with sterile equipment, to prevent contamination and avoid subsequent infection. Therefore, the ability of the IVR practice to help the students to familiarize themselves with the steps involved in the procedure regarding the placement of Foley catheters in female patients is one of its most important benefits.

#### 3.5.3. Understanding Clinical Situations before Internship

Some of the students also mentioned that using VR to practice helped them attain a more thorough understanding of actual clinical situations. Students made the following statements: “It has greater influence before our internship. We get to know about clinical situations ahead of time” (Student 4); “It was like seeing what we will see in a clinical setting, so we won’t be shocked when seeing the real thing when we enter an actual clinical setting” (Student 8); and “I think we can practice what we will encounter in a clinical setting in the VR world ahead of time. I think it’s not bad” (Student 9). Many of the students believed that experiencing the clinical scenarios before starting their internships would help them to succeed during their internships.

#### 3.5.4. Understanding the Applications of IVR Technology in Medicine

Some of the students also mentioned that IVR-based practice made them more aware of the applications of technology in medicine. For example, students made the following statements: “(The school) prepared ahead of time and let us know that VR and many other artificial intelligence–related things will come” (Student 1); “(VR) broadened my vision!” (Student 5); and “VR is able to connect things that are related. Through it, I can see a lot of things. Now, it is applied in many areas” (Student 4). Some of the students felt that through the IVR practice, the school intended to make the students aware of the diverse applications of technology. They felt that the nursing education curriculum had been planned ahead of time to help students understand the various applications of VR and artificial intelligence.

### 3.6. Critical Thinking

Some of the students reported that using IVR to practice how to place Foley catheters in female patients was surprising, fun, and effective in helping them learn. Moreover, the experience inspired them to reflect on the use of VR in nursing education. For example, students made the following statements: “Regarding the technical aspect, machines can replace practice. However, I feel that the other role of nurses is to provide care and companionship” (Student 8); “(VR) is cold. For example, in our basic nursing theory course, we were taught to pay attention to the patients’ reactions and use communication skills such as repeating (what the patients said). These were not applied [in the VR practice]” (Student 12); “During the IVR practice, we should care about the patients. I feel I couldn’t engage in much communication” (Student 5); “I feel we, as nurses, sometimes should provide patients with strength” (Student 8); and “In principle, (VR) will not make you feel warmth, and it will not make the patient feel empathy” (Student 3). As reflected in these comments, the students thought critically about the IVR practice and were concerned about its ability to help them practice providing patient-centered care.

## 4. Discussion

According to the questionnaire survey and the focus group interview, over half of the students believed that the IVR training helped them overcome the difficulties they encountered in learning how to insert Foley catheters inside female patients (58.6%) and memorize the operational steps involved in the procedure regarding the placement of Foley catheters in female patients (62.8%). Furthermore, over half (52%) of the students agreed with the statement, “Using VR to practice makes me feel as though I were in a clinical setting.” Some of the students mentioned that although they used VR to practice only the technique regarding how to place Foley catheters in female patients in the present study, in the future, VR could be used to help nursing students to practice providing care to patients with acute and severe symptoms, thereby helping students to feel less overwhelmed when starting a clinical internship or subsequent work. Student 6 said, “I wonder if VR can be integrated with lessons on addressing acute and severe symptoms or navigating the operating room. If I can experience the image of the trauma ahead of time, when I work in a clinical setting, I will not be too scared to know what to do.” Our course observations revealed that the students were diligent in using VR to practice the technique. Their reflections on the IVR practice, therefore, provide valuable insights into the use of VR in nursing education. According to one study, using VR to practice a technique positively affects the nursing students’ critical thinking abilities [24]. Another study revealed that by enabling students to undergo innovative simulations and training, VR can increase students’ confidence [25]. Moreover, in the present study, over 90% (39) of the students maintained that using VR to practice how to place Foley catheters in female patients was convenient. Practicing the technique using the model involved many physical objects, such as sterilized cotton balls, urinary catheters, urine bags, and catheterization kits, which must be disposed of after the actual procedure, resulting in medical waste. Most of the students exhibited a positive attitude toward using VR to learn additional techniques in the future. According to the focus group interview, the IVR practice was a pleasant learning experience. Most of the students reported that IVR was novel and interesting. In another study in which nursing students were interviewed on the use of VR in nursing education, the researchers determined that VR effectively helped the students to learn skills by complementing conventional teaching methods with novel and intriguing content [26]. In the present study, students mentioned that using VR to practice how to place Foley catheters in female patients had a substantial impact on them because it helped them to gain a more thorough understanding of the technique before they started interning or working at the hospital. Student 4 said, “If I can learn the concept ahead of time, in the future, I will be more willing to use VR,” and Student 7 said, “The school gave me the feeling that it had been preparing ahead of time to let us know about VR. In the future, many other artificial intelligence products may be involved.” VR helped the students to effectively familiarize themselves with the operational steps involved in the procedure regarding the placement of Foley catheters in female patients and enabled the students to explore a clinical setting within the virtual environment. According to another study, the use of VR has become increasingly common in various fields. In addition to its applications in education, VR is helpful in the cognitive assessment of mental disorders and mood disorders [27] and in helping people recover from motion sickness [28]. In another study evaluating the use of mobile VR in operating-room nursing education, the nursing students who had completed a mobile VR–based course outperformed those who had not [29]. The results of the aforementioned study and of the present study may serve as a reference for the application of VR in nurse-training courses, especially those focused on specific clinical skills.

In addition to feeling that the IVR practice was effective in helping them learn, the students also reflected critically on the IVR practice. Although the students felt that using VR made them feel as though they were in an actual clinical setting, which helped them understand the operational steps involved in the procedure regarding the placement of Foley catheters in female patients, as well as the applications of technology in medical education, they also felt that clinical care should involve communication with patients, empathy, and compassion. They thought that the IVR training should have reflected the role of nurses as care providers. In the questionnaire, 18 of the participants (41.4%) indicated that their experience in learning how to insert in-dwelling urinary catheters through VR training was a mediocre experience. In the focus group interview, the participants expressed their feeling that the capacity for expressing empathy, concerns, and companionship was lacking in VR interactions. This demonstrates the participants’ ability to express their opinions on VR and describe its pros and cons. One study of the effects of VR on the knowledge, attitudes, and behaviors of healthcare workers revealed that VR had considerable benefits related to the “affective domain of learning,” especially in promoting empathy. Increased empathy helped improve the attitudes and behaviors of the participants in caring for patients, thereby helping them to provide a better quality of care [30]. The results of this study demonstrated the participants’ ability to think critically about using VR. “We should learn to express empathy to patients in immersive VR training, but I do not feel we can communicate further in it” (Participant 5). The participants expressed concerns regarding learning how to provide empathic care. However, in the present study, many of the students reported that the simulated interaction in the VR practice did not require them to express empathy, compassion, or other principles of patient-centered care. In the future, principles of patient-centered care should be integrated into VR-based courses on clinical skills. In terms of equality of education, although only one participant expressed discomfort in using VR, this problem must be addressed in future studies. This study conducted focus-group interviews to collect students’ VR usage experiences, and the responses provided by the students were analyzed. Some of the students had previous VR usage experiences, and their satisfaction with VR varied. This signified that the students’ responses may have been biased, which was a limitation of this study.

## 5. Conclusions

The participants in this study, who were students in a postbaccalaureate nursing program, were satisfied with the experience of using IVR to learn how to place Foley catheters in female patients. They considered the IVR training to be convenient and reported that they would like to continue using IVR to practice clinical skills in the future. The IVR practice had multiple positive effects on the students, which are summarized as follows. (1) The students had a pleasurable learning experience. Most of the students who had not used VR in the past did not reject the IVR training; rather, they felt that it was surprising and interesting. (2) Using VR to practice made the students feel as though they were actually in a clinical setting. They felt a sense of presence and could interact with the patient in the simulation. (3) Using VR to practice effectively helped the students overcome their difficulties in learning the procedure, specifically by helping them to memorize the operational steps involved in learning how to place Foley catheters in female patients. (4) The students reported that the IVR practice made them feel more prepared for clinical practice and that they expected to feel shocked by actual clinical scenarios upon entering a hospital setting. (5) In addition, by helping the students to learn how to place Foley catheters in female patients, the IVR training encouraged the students to reflect critically on the use of technology in nursing education and provided them with a new perspective on the role of nurses as care providers. The students argued that VR-based training courses should integrate features that require students to express empathy, compassion, and other principles of patient-centered care.

## Figures and Tables

**Table 1:** Research process.

Stage	Content
Pre-teaching	Key points about how to place Foley catheters in female patients (5-min digital teaching materials). VR instructions (5-min digital teaching materials).
Teaching	In class, the teacher demonstrated how to place Foley catheters in female patients by using VR. The students were divided into seven groups of 5–6 students to practice the technique by using VR. The researchers and teaching assistants moved among the groups to observe and guide the students when practicing. When a group member was practicing using VR, the other group members observed the projected screen simultaneously. The VR case scenario was used in class for the teacher and students to discuss.
Post-teaching	Each student was required to practice the technique with the VR system at least twice. After the VR practice, the students completed the learning satisfaction questionnaires. After all the group members finished practicing, focus group discussions were conducted to gather the students’ feedback on how to modify the VR training in the future.

**Table 2:** Learning experience summary table.

Themes	Categories
Pleasurable experience	Fun
2. Effective learning	2. Experiencing clinical scenarios
	3. Memorizing steps of the technique
	4. Understanding clinical situations before working at the hospital
3. Critical thinking	5. Inability to reflect the role of nurses as care providers

## Data Availability

Not applicable.
